# From Crisis Response to Lasting Transformation: Five-Year Insights from the Implementation of Telemedicine in Neurosurgical Care During COVID-19

**DOI:** 10.3390/healthcare13222939

**Published:** 2025-11-17

**Authors:** Olga Mateo-Sierra, Elena Romero-Cumbreras, Estela García-Llorente, Sofía Rubín-Alduán

**Affiliations:** 1 Department of Neurosurgery, Gregorio Marañón University Hospital, C/Dr Esquerdo 46, 28007 Madrid, Spain; 2 Gregorio Marañón Research Institute, Gregorio Marañón University General Hospital, C/Dr Esquerdo 46, 28007 Madrid, Spain; 3 Department of Surgery, Medicine School, Complutense University of Madrid, 28040 Madrid, Spain; 4 Department of Radiology, Infanta Leonor University Hospital, Avda Gran Vía del Este, 80, Vallecas, 28031 Madrid, Spain; 5 Department of Psychiatry, Ramón y Cajal University Hospital, M-607, Km9,100, Fuencarral-El Pardo, 28034 Madrid, Spain; 6 Department of Anesthesiology, Ramón y Cajal University Hospital, M-607, Km9,100, Fuencarral-El Pardo, 28034 Madrid, Spain

**Keywords:** COVID-19, telemedicine, neurosurgery, continuity of care, healthcare resilience, outpatient services, Madrid

## Abstract

**Background:** The COVID-19 pandemic profoundly disrupted healthcare systems worldwide, compelling rapid adaptation of clinical workflows and accelerating the integration of telemedicine. **Objective:** This study evaluates the implementation of telemedicine in neurosurgical outpatient care at a tertiary referral hospital in Madrid during the first epidemic wave (March–May 2020) and explores its long-term significance five years later. **Methods:** A retrospective observational analysis including 5175 neurosurgical outpatient consultations was conducted, comparing the first epidemic wave of COVID-19 (2070 teleconsultations) with the equivalent period in 2019 (3105 in-person visits). Demographic, clinical, and procedural data were analyzed, including six-month follow-up outcomes. Univariate and multivariate analyses were performed to identify factors associated with teleconsultation use and follow-up delay. **Results:** The total number of consultations decreased by 33% compared to the pre-pandemic year. In May 2020, teleconsultations represented more than 70% of all visits. Continuity of care was preserved (follow-up adherence >80%), and missed appointments declined to zero. Cranial and oncological pathologies were prioritized, while degenerative and benign cases were largely deferred. Teleconsultation independently predicted delayed six-month follow-up (aOR 1.9, 95% CI 1.3–2.8, *p* = 0.002) and a lower likelihood of surgical indication (aOR 0.4, 95% CI 0.2–0.7, *p* = 0.004). Despite these differences, remote care ensured accessibility, safety, and clinical continuity under extreme healthcare system strain. **Five years perspective**: In addition to these early outcomes, the study describes the sustained integration of telemedicine during the subsequent five years, illustrating how this model became permanently embedded in routine neurosurgical practice in this center. **Conclusions:** This study represents one of the earliest structured telemedicine experiences in Spanish neurosurgery. The rapid adaptation of the Hospital General Universitario Gregorio Marañón ensured care continuity during the pandemic and catalyzed the lasting adoption of hybrid models that enhance accessibility, safety, efficiency, and healthcare system resilience.

## 1. Introduction

The coronavirus disease 2019 (COVID-19) pandemic, caused by the severe acute respiratory syndrome coronavirus 2 (SARS-CoV-2), was first identified in Wuhan, China, in December 2019, when the initial cluster of pneumonia cases was reported. Chinese authorities notified the World Health Organization (WHO) on 7 January 2020, and the disease was declared a Public Health Emergency of International Concern on 30 January, followed by a global pandemic on 11 March 2020 [1,2]. From that point onward, infection numbers increased exponentially—first in China and rapidly across the world [3].

In Spain, the first confirmed case was reported on 31 January 2020. Within weeks, the country experienced one of the most severe outbreaks in Europe. The Community of Madrid became the epicenter, with more than 64,000 confirmed cases by May 2020 [4,5]. The Hospital General Universitario Gregorio Marañón (HGUGM), one of Madrid’s largest tertiary referral centers, was among the institutions most affected, facing critical overload during the first epidemic wave.

The pandemic’s rapid escalation profoundly disrupted healthcare practice. Hospitals across Spain and worldwide were forced to reorganize clinical workflows and redistribute staff and infrastructure. Non-urgent surgeries and outpatient activity were suspended, while healthcare professionals were redeployed to COVID-19 units and intensive care wards [6,7]. This abrupt transformation highlighted the urgent need for alternative mechanisms to ensure care continuity while minimizing exposure risk.

Telemedicine, although conceptually established since the mid-20th century and introduced into neurosurgery in the 1990s [8], had remained marginal before 2020. The COVID-19 crisis acted as a powerful catalyst for its adoption, dismantling long-standing barriers such as insufficient digital infrastructure, lack of training, and regulatory uncertainty. Early reports confirmed its potential to preserve continuity of care, reduce unnecessary hospital visits, and mitigate transmission risk [9,10,11].

Its limited pre-pandemic adoption reflected a combination of structural, regulatory, and cultural barriers. The absence of standardized reimbursement frameworks, uneven digital infrastructures, and concerns regarding data protection and medico-legal liability hindered large-scale implementation and cultural acceptance [12,13]. Furthermore, many clinicians also regarded remote consultation as inadequate for specialties requiring physical examination or procedural planning. Comparative studies across medical fields highlighted both the advantages and limitations of telemedicine, showing comparable diagnostic accuracy and high patient satisfaction, but raising questions about a continuity of care, cost effectiveness, and equity [11,14,15]. However, few analyses have explored these dynamics in surgical disciplines, and virtually none have explored their long-term institutional integration.

Neurosurgery was among the most affected specialties, due to operating room restrictions and personnel reallocation to COVID-19 care, which led to the suspension of elective and outpatient procedures [16,17]. Similar telemedicine initiatives emerged worldwide to sustain patient follow-up during lockdown [18,19,20]. In Spain, the transition occurred swiftly and organically, driven by institutional necessity and supported by regional digital health initiatives [21,22].

At HGUGM, the Department of Neurosurgery transitioned its outpatient activity to remote telephone consultations within days, ensuring uninterrupted patient care. What began as an emergency adaptation evolved into a stable, scalable digital workflow that persists five years later. Similar trends have been observed in other Spanish hospitals across multiple specialties, where telemedicine remains integrated into hybrid care models [23,24,25,26,27].

This study describes the rapid implementation and clinical outcomes of telemedicine in neurosurgical outpatient care at HGUGM during the first wave of COVID-19. It provides quantitative evidence of care adaptation, examines univariate and multivariate factors associated with follow-up and surgical indication, and contextualizes the long-term role of telemedicine in consolidating a hybrid model of neurosurgical practice in Spain.

Spain was among the European countries most severely affected during the first wave, with early and prolonged restrictions on elective hospital activity. Neurosurgery, as a high-complexity specialty heavily dependent on in-person evaluation, represented a critical test case for assessing the feasibility of telemedicine in surgical care. Moreover, HGUGM was one of the first tertiary centers in Spain to implement a structured telemedicine program under emergency conditions. Beyond the quantitative assessment of the first pandemic wave and six-month outcomes, a five-year institutional perspective provides qualitative insight into how telemedicine transitioned from an emergency measure to a stable component of outpatient neurosurgical practice.

## 2. Materials and Methods

This retrospective observational study evaluated the impact of the COVID-19 pandemic on neurosurgical outpatient activity and analyzed the rapid implementation of telemedicine in a tertiary referral hospital. It was conducted at the Department of Neurosurgery, Hospital General Universitario Gregorio Marañón (HGUGM), Madrid, Spain—one of the largest tertiary centers in the region and among those most affected during the first epidemic wave of COVID-19.

Two cohorts were compared the pre-pandemic cohort (2019), including 3105 patients attended in person between 1 March and 31 May 2019, and the pandemic cohort (2020), comprising 2070 patients who received telemedicine consultations during the same period of 2020. In total, 5175 outpatient consultations were analyzed. In both groups, six-month follow-up data were reviewed to assess continuity of care, adherence to recommended reviews, and potential delays in clinical management.

Eligible participants were adult patients (≥18 years) scheduled and evaluated in the Neurosurgery Outpatient Department during either the first wave of the pandemic (2 March–29 May 2020) or the corresponding period in 2019. Exclusion criteria included incomplete clinical documentation, absence of follow-up data, or missing initial or six-month consultations. Data were retrieved from the hospital’s electronic medical record system (HCIS^®^, DXC Technology, Tysons, VA, USA) and compiled into a structured database specifically developed for this study. The dataset included demographic, clinical, and temporal variables for all patients scheduled in both periods, allowing a comprehensive comparison of outpatient activity, consultation modality (in-person vs. teleconsultation), pathology type, and clinical outcomes. Longitudinal follow-up data up to six months after the initial consultation enabled evaluation of care continuity, outcomes, and timing of re-evaluation.

This study evaluated demographic, clinical, and procedural data collected from the electronic medical record (HCIS) (Table 1, Table 2 and Table 3).

Primary outcomes were defined as continuity of care, measured by adherence to six-month follow-up, and surgical indication status. These endpoints were chosen to reflect the core dimensions of outpatient neurosurgical performance: patient retention and clinical decision making. Secondary variables included pathology type (cranial, spinal, or other; vascular, traumatic, degenerative and tumoral), diagnostic-test requests, and consultation characteristics (teleconsultation vs. in-person, by month). Six-month follow-up adequacy was categorized as on time (within the physician-recommended period), delayed (beyond the recommended time), no-show, or other. For each patient, the actual time to next recorded visit was compared with the advised interval. Follow-up was considered on-time when the appointment occurred with the prescribed period or up to one month later and delayed when the interval exceeded this threshold. Non-attendance was defined as a scheduled outpatient consultation not completed or cancelled without rescheduling, according to hospital’s digital appointment registry (HCIS). Clinical deterioration or misdiagnosis was assessed by reviewing follow-up notes and diagnostic concordance at six months. All variables were analyzed for both cohorts, and descriptive and comparative summaries were compiled in tables (Table 1, Table 2 and Table 3).

Descriptive statistics were used to summarize all data. Continuous variables were expressed as mean ± standard deviation (SD) when normally distributed or as median and interquartile range (IQR) otherwise, while categorical variables were expressed as absolute and relative frequencies (%). Comparisons between qualitative variables were performed using Pearson’s Chi-square test or Fisher’s exact test when appropriate, and quantitative comparisons were conducted with Student’s *t*-test for parametric data. A univariate analysis was performed to explore associations between clinical variables (pathology type, consultation outcome, and follow-up adequacy) and consultation modality (in-person vs. telemedicine), calculating odds ratios (OR) and 95% confidence intervals (CI). A multivariate logistic regression model was subsequently applied based on clinical relevance and significance in univariate analysis (*p* < 0.10). The final model included age, sex, pathology type, consultation modality (teleconsultation vs. in-person), and the primary outcomes (follow-up adherence and surgical indication), as these were considered the main determinants of outpatient neurosurgical workflow during the study period. Adjusted odds ratios (aOR) with 95% CI were reported. All analyses were conducted using IBM SPSS Statistics, version 25.0 (IBM Corp., Armonk, NY, USA), and statistical significance was defined as *p* < 0.05.

This study received approval from the Ethics Committee of Hospital General Universitario Gregorio Marañón (HGUGM) (code TELEMATIC-NS-COVID19) and was conducted in accordance with the principles of the Declaration of Helsinki. Given its retrospective design, the requirement for informed consent was waived. All patient data were anonymized prior to analysis to ensure confidentiality and compliance with the European General Data Protection Regulation (EU GDPR 2016/679) and the Spanish Organic Law 3/2018 on Data Protection and Guarantee of Digital Rights.

## 3. Results

### 3.1. Epidemiological Context: National and Regional Infection Data

During the first epidemic wave (March–May 2020), Spain experienced one of the highest COVID-19 incidence and mortality rates in Europe, leading to a nationwide suspension of elective surgical activity. The Community of Madrid accounted for nearly one-third of national hospital admissions, severely affecting tertiary centers such as HGUGM. This context explains the abrupt reorganization of outpatient neurosurgical services and the rapid implementation of telemedicine as an alternative mode of care [4].

### 3.2. Patient Demographics and Consultation Distribution

A total of 5175 neurosurgical outpatients were included—3105 patients (60%) attended in 2019 (pre-pandemic cohort) and 2070 (40%) in 2020 (pandemic cohort), totaling 5175 (Table 2). No statistically significant demographic differences were found between cohorts (*p* > 0.05). The mean age was 59 ± 15 years (2019: 59; 2020: 58), and sex distribution was similar (56% female, 44% male).

**Figure 1:**
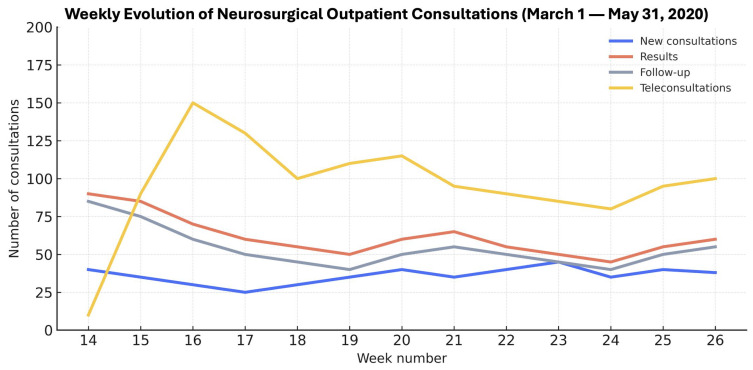
Weekly evolution of neurosurgical outpatient activity during the first epidemic wave (March–May 2020). X-axis = week number for each study year. The sharp increase in teleconsultations reflects the rapid transition to remote care at the onset of the pandemic.

Monthly outpatient volume declined sharply during 2020, paralleling the epidemiological curve of the pandemic (Figure 1). Consultations fell from 981 in March to 611 in April and 478 in May (*p* < 0.001), showing a clear inverse relationship between infection surge and hospital attendance.

### 3.3. Consultation Types and Attendance

**Figure 2:**
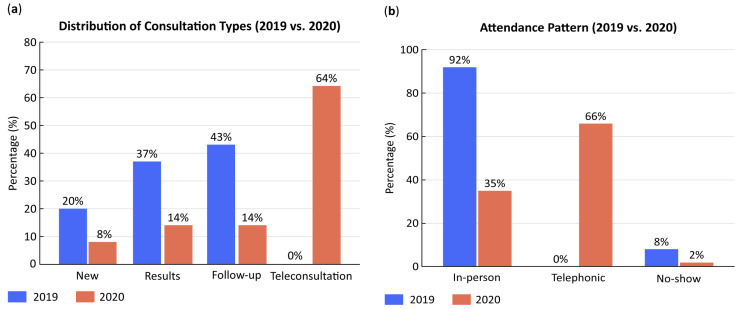
(**a**) Comparison of outpatient consultation categories (new, results, and follow-up) during March–May 2019 and the same months in 2020. Teleconsultations, nonexistent in 2019, represented over 70% of visits by May 2020, replacing most in-person appointments. (**b**) Comparison of attendance pattern. In 2020, not only did the type of consultation change, shifting to telephone visits, but the no-show rate also decreased.

In 2019, the distribution of consultation types was consistent: new visits (21%), results (37%), and follow-ups (43%), all performed in person. In 2020, however, consultation dynamics changed dramatically (Table 3; Figure 2).

New consultations decreased from 14% in March to 1% in May (*p* < 0.001); results visits declined from 24% to 9% (*p* < 0.001); and follow-ups dropped from 21% to 14% (*p* < 0.001). In contrast, teleconsultations—non-existent in 2019—rapidly became predominant, representing 74% of total visits in April and 77% in May (*p* < 0.001).

Attendance patterns mirrored this shift. Face-to-face visits fell from 56% in March to 23% in May, while telephone consultations increased from 39% to 77%. Non-attendance, which had averaged 8% in 2019, declined to 0% by May 2020, indicating improved accessibility and compliance through remote care.

### 3.4. Distribution by Pathology Type and Disease Category

**Figure 3:**
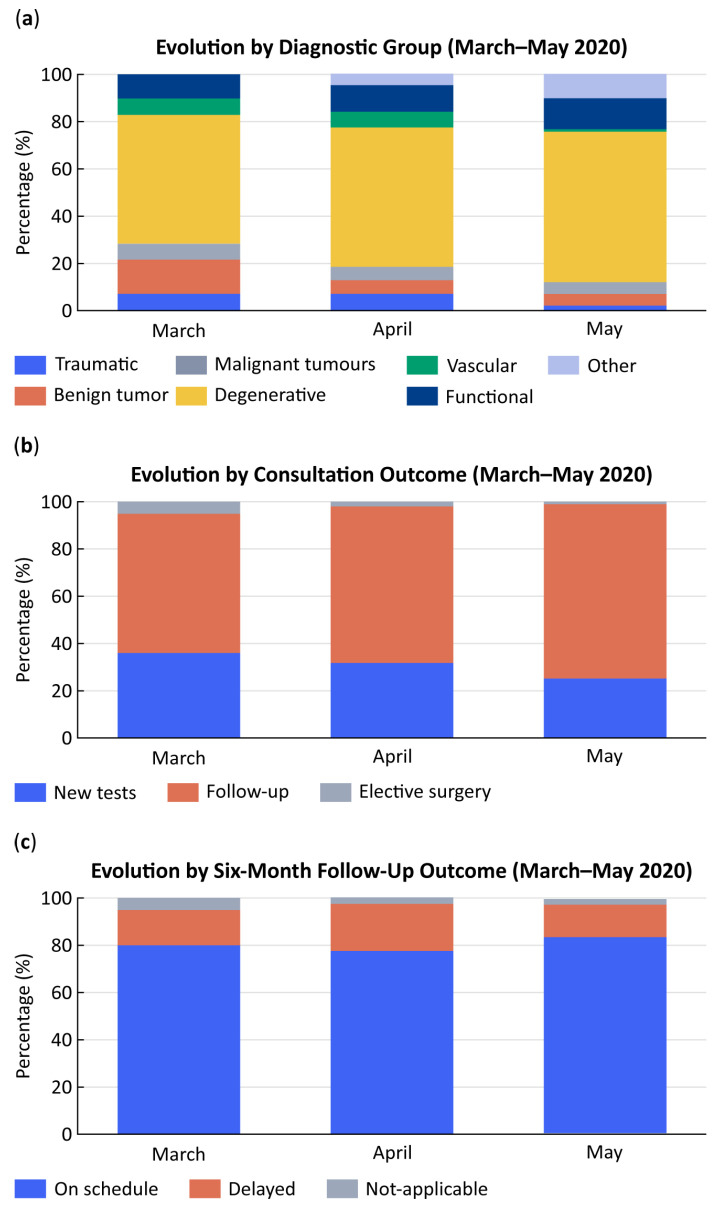
Attendance pattern by year and consultation modality. Monthly evolution of neurosurgical outpatient activity at Hospital General Universitario Gregorio Marañón (HGUGM) during the first wave of the COVID-19 pandemic (March–May 2020): (**a**) distribution by diagnostic group, (**b**) consultation outcomes, and (**c**) six-month follow-up results.

Analysis of clinical diagnoses revealed major shifts in case mix (Table 4; Figure 3a). All diagnostic categories decreased markedly across March–May 2020, consistent with the suspension of elective activity. Cranial pathologies dropped from 58% in March to 11% in May (*p* < 0.001), and spinal disorders from 56% to 12% (*p* < 0.001). Other pathologies—such as peripheral nerve, vascular, or functional disorders—fell from 74% to 14% (*p* < 0.001).

When grouped by oncological status, non-tumoral conditions declined from 46% in March to 25% in May (*p* < 0.001), and tumoral cases from 60% to 11% (*p* < 0.001). Within the tumoral subgroup, malignant lesions were preserved in relatively higher proportion compared with benign or degenerative disorders, reflecting clinical prioritization of urgent and high-risk cases.

It is important to note that acute neurosurgical conditions—including cranial trauma, hydrocephalus, and high-grade tumors—are typically managed through the emergency department rather than outpatient clinics. Therefore, their lower representation in this cohort does not indicate reduced care provision; rather it reflects the institution’s established triage and referral pathways.

### 3.5. Consultation Outcomes

Marked differences were observed in consultation outcomes (Figure 3b). Requests for diagnostic tests and surgical indications decreased significantly (*p* < 0.001), due to restricted access to imaging and operating rooms. Conversely, follow-up reviews increased proportionally, representing an adaptive response to maintain patient surveillance with limited hospital resources. Non-attendance dropped to zero, according to hospital scheduling records that automatically log all missed or cancelled appointments.

Surgical indications declined dramatically—from 74% in March to 3% in May (*p* < 0.001)—confirming that only urgent cases, mainly malignant tumors and acute cerebrospinal fluid disorders, were prioritized during lockdown.

### 3.6. Six-Month Follow-Up

At six months, adherence to follow-up remained high, with most patients reviewed within the recommended period (Figure 3c). On-time reviews accounted for 81% in March and 83% in May, while delayed visits represented 15–20% of the total.

Delay rates did not differ significantly by month (*p* > 0.05). However, teleconsultations were independently associated with a higher likelihood of delayed six-month follow-up compared with in-person visits (aOR = 1.9; 95% CI 1.3–2.8; *p* = 0.002). Despite this difference, overall adequacy of follow-up remained high (>80%), confirming preserved clinical continuity under crisis conditions.

No cases of clinical deterioration or diagnostic discordance were identified in the review of follow-up visits and electronic medical records conducted at six months postconsultation.

This pattern likely reflects the broader epidemiological context: as the pandemic progressed, telemedicine became the only viable form of consultation, naturally concentrating follow-up volume in remote modalities Nevertheless, remote care effectively prevented patient loss to follow-up and maintained clinical continuity under extreme healthcare pressure.

### 3.7. Univariate and Multivariate Analysis

The univariate analysis demonstrated significant associations between the pandemic period, consultation mode, and clinical outcomes (Table 5). Tumoral (OR 0.6; *p* < 0.001) degenerative (OR 0.5; *p* < 0.001); and vascular (OR 0.3; *p* < 0.001) pathologies significantly decreased during the pandemic months. Requests for diagnostic tests (OR 0.5; *p* < 0.001) and surgical indications (OR 0.2; *p* = < 0.001) also declined, whereas follow-up visits increased (OR 0.7; *p* < 0.001). Delayed six-month reviews were twice as likely after teleconsultation (OR 2.0; *p* < 0.001).

In the multivariate model (Table 5), teleconsultation independently predicted delayed follow-up (aOR 1.9, 95% CI 1.3–2.8; *p* = 0.002) and a lower likelihood of surgical indication (aOR 0.4, 95% CI 0.2–0.7; *p* = 0.004). Tumoral pathology remained the strongest predictor of surgical decision (aOR 3.6, 95% CI 2.1–5.9; *p* < 0.001), confirming that oncological status was the primary determinant of operative management during the pandemic.

## 4. Discussion

Although the first wave of the COVID-19 pandemic occurred five years ago, the present study remains highly relevant. It documents one of the earliest structured experiences of telemedicine implementation in neurosurgical care in Spain, marking a turning point in the digital transformation of clinical practice [24]. Beyond its historical significance, it provides a longitudinal perspective on how crisis-driven innovations evolved into sustainable care models. The lessons learned—rapid digital adaptation, hybrid patient management, and enhanced accessibility—are directly applicable to current healthcare priorities, including resilience, efficiency, and equity in digital medicine.

The abrupt onset of the pandemic in early 2020 led to an unprecedented collapse of hospital systems in Madrid, one of the hardest-hit regions in Europe [28,29]. Over 64,000 infections were recorded during the first wave, forcing hospitals such as the Hospital General Universitario Gregorio Marañón (HGUGM) to reorganize virtually overnight [30,31]. As reported by international studies, this situation prompted widespread disruption of elective and outpatient activity in neurosurgery [17,18,32]. In this context, telemedicine emerged as the only feasible mechanism to ensure care continuity, patient safety, and infection control.

At HGUGM, the introduction of telephone-based teleconsultation was achieved within days, enabling more than 70% of neurosurgical visits to be performed remotely by May 2020. Despite an approximately 33% decrease in total consultations, follow-up compliance remained high (>80%), and non-attendance rates dropped to nearly zero. This demonstrates that telemedicine effectively sustained patient engagement, while also confirming its feasibility and clinical value in a highly specialized surgical setting. Comparable experiences were described across international centers: De Biase et al. (2020) reported a 55% conversion to telemedicine in a U.S. tertiary center [18], and Taha et al. (2021) in Australia observed similar adoption rates [33]. In Europe, Apostolopoulou et al. (2022) described that over 80% of pediatric neurosurgical visits were conducted remotely during national lockdowns [34]. The parallel evolution of these models underscores the universal capacity of telemedicine to maintain neurosurgical care during large-scale health crises.

Our findings reveal notable shifts in clinical case mix and care dynamics. Degenerative and benign spinal pathologies—normally predominant in neurosurgical outpatient practice—declined sharply, while malignant and urgent cranial cases were prioritized. It is important to note that acute trauma and emergency cranial cases are typically evaluated directly through emergency departments rather than outpatient clinics, both before and during the pandemic, explaining their lower representation in this study cohort.

This targeted deferral of degenerative and benign cases inevitable introduced a degree of selection bias in the comparative outcomes. The 2020 cohort therefore represented a population with higher clinical acuity, predominantly oncological and urgent cranial conditions, while the 2019 cohort included a broader spectrum of elective pathologies. As a result, differences in follow-up and surgical indication rates should be interpreted within this context of case prioritization rather than as intrinsic effects of telemedicine itself. This selective maintenance of high-risk pathologies reflects rational triage strategies consistent with international guidelines and early recommendations from the COVIDSurg Collaborative (2020) [35].

At the same time, restrictions on imaging and surgical capacity limited diagnostic and operative throughput. The significant reduction in surgical indications (*p* < 0.001) mirrors global trends reported by Kessler et al. (2020) [36] and Jean et al. (2020) [37], who documented the near-total suspension of non-urgent procedures during this phase. Both system-level and physician-level factors contributed to these declines. Operating-room and imaging restrictions imposed by institutional contingency plans represented a major resource-driven limitation. In parallel, neurosurgeons applied clinical triage to postpone low-risk or elective cases, in accordance with national and international recommendations. The observed decrease in diagnostic test requests and surgical indications therefore reflects a combined effect of capacity constraints and deliberate prioritization rather than a reduction in clinical demand.

The six-month follow-up data further confirm that remote consultation preserved long-term care continuity. Although delayed reviews were twice as frequent among teleconsultation patients (20.9% vs. 10.4%), overall adequacy remained comparable (77% vs. 83%). This finding must be interpreted in context: as the pandemic advanced, teleconsultation became the dominant, and often the only, available mode of follow-up. Thus, the apparent delay reflects system-wide disruption rather than inefficiency of the telemedicine model itself. On the contrary, remote care allowed timely monitoring in circumstances where face-to-face evaluation was unfeasible, highlighting its role in mitigating clinical risk.

Teleconsultation was independently associated with delayed six-month follow-up, although overall adherence exceeded 80%. This suggests that remote consultations may extend review intervals slightly, likely reflecting reduced access to diagnostic resources during the first pandemic wave rather than intrinsic limitations of telemedicine.

These figures align with those from Eichberg et al. (2020) [38] and Vogt et al. (2022) [10], who demonstrated that patient satisfaction, adherence, and safety outcomes in telemedicine neurosurgical follow-up were equivalent to in-person models. Importantly, in our cohort, no cases of clinical deterioration or diagnostic omission were reported due to teleconsultation, underscoring its reliability for stable or postoperative patients.

From a broader perspective, the experience at HGUGM was consistent with the evidence generated by the COVIDSurg Collaborative on perioperative risk, surgical delay, and postoperative outcomes during the pandemic [36,39]. These international data contextualize the ethical and logistical dilemmas faced by surgical teams: balancing infection risk, resource limitations, and the need to continue essential oncological or urgent interventions. In this regard, the telemedicine framework developed at HGUGM represented a practical and safe adaptation that preserved access while minimizing exposure.

Telemedicine also introduced long-term clinical and socioeconomic advantages. By reducing unnecessary hospital visits, it lowered patient travel burden, optimized scheduling, and facilitated multidisciplinary coordination—particularly valuable for chronic or postoperative neurosurgical cases. Moreover, it contributed to reducing nosocomial infection risk, not only for SARS-CoV-2 but also for other transmissible diseases. Economic analyses have demonstrated that telemedicine models improve efficiency and reduce healthcare expenditure [10,12], findings that are consistent with the operational experience at HGUGM.

While this study quantitatively focused on the initial pandemic period (March–May 2020) and six-month outcomes, a five-year institutional follow-up confirmed the sustained integration of telemedicine into routine neurosurgical workflows. This longitudinal perspective complements the early outcome data, offering contextual “five-year insights” into the durability and structural impact of telemedicine at HGUGM.

From an institutional perspective, the introduction of telemedicine at HGUGM triggered a long-term reorganization that extended beyond the acute pandemic phase. Five years later, teleconsultation remains integrated into routine neurosurgical workflows—especially for postoperative reviews, communication of results, and follow-up of stable patients. Similar patterns of persistence in telemedicine use have been documented in Spanish clinical settings, supporting the notion that remote consultation models have achieved sustainability and acceptance across specialties. In line with institutional strategies, the Hospital General Universitario Gregorio Marañón and other hospitals in the Madrid Regional Health Service (SERMAS) have maintained teleconsultation systems as part of their post-pandemic digital care plans, citing improved accessibility, reduced waiting times, and enhanced infection control [23,40].

Building on this experience, a structured hybrid model can be proposed for neurosurgical outpatient care. In-person consultations remain essential for initial evaluation, neurological examinations, surgical decision-making, and the discussion of new imaging results, where physical assessment and direct communication are critical. Teleconsultations, in contrast, are ideally suited for postoperative reviews, medical adjustments, and long-term follow-up of stable or chronic conditions, allowing patients to avoid unnecessary travel and facilitating multidisciplinary coordination. Intermediate scenarios, such as reassessment of new symptoms or ambiguous imaging findings, should follow a flexible approach, with remote triage guiding timely face-to-face visits when needed. This framework reflects the practical organization currently adopted at HGUGM and may serve as a transferable model for other tertiary centers seeking to balance efficiency, accessibility, and clinical safety in neurosurgical practice.

Altogether, this experience demonstrates that even under conditions of healthcare system collapse, rapid digital transformation is possible. Telemedicine enabled uninterrupted neurosurgical care, preserved patient safety, and laid the foundation for hybrid models that continue to define patient-centered care in modern healthcare systems.

## 5. Highlights

This study provides one of the earliest structured evaluations of telemedicine implementation in neurosurgical outpatient care in Spain. During the first wave of the COVID-19 pandemic, more than 70% of neurosurgical consultations were conducted remotely while maintaining follow-up adherence above 80%. Teleconsultation significantly reduced non-attendance and ensured continuity of care despite the collapse of in-person healthcare systems. The analysis revealed a sharp decline in degenerative and benign pathologies, while urgent and malignant cases were prioritized according to clinical severity. Five years later, telemedicine remains integrated into routine neurosurgical workflows, demonstrating its long-term sustainability and contribution to the development of modern hybrid healthcare models that enhance accessibility, efficiency, and resilience.

## 6. Strengths and Limitations

This study presents several methodological and contextual strengths. It includes a large, well-defined cohort with a direct year-to-year comparison (2019 vs. 2020) within a major tertiary neurosurgical center. The analysis integrates both univariate and multivariate models, providing quantitative insight into how clinical workflows and patient outcomes adapted to the abrupt digital transformation prompted by the COVID-19 pandemic. The inclusion of six-month follow-up data strengthens the evaluation of care continuity and highlights the long-term reliability of teleconsultation. Moreover, this work offers one of the earliest structured assessments of neurosurgical telemedicine implementation in Spain, contributing valuable evidence to the ongoing digital transformation of healthcare systems.

Nevertheless, several limitations should be acknowledged. The retrospective and single-center design may limit generalizability, and patient-reported outcomes and satisfaction metrics were not collected, restricting the assessment of user acceptance of telemedicine. Diagnostic concordance was also not formally measured. The exclusive use of telephone-based teleconsultations restricted direct imaging review and neurological examination, potentially affecting diagnostic precision in complex or newly presenting cases. Furthermore, because elective and degenerative pathologies were largely deferred during the lockdown, the 2020 cohort primarily comprised urgent and oncological cases, introducing a potential selection bias that could influence comparative follow-up and surgical indication outcomes. Additionally, disparities in access to technology—particularly among elderly or socioeconomically disadvantaged patients—may have influenced the adoption of remote care. These challenges underscore the need for complementary hybrid models that combine digital innovation with equitable access and clinical rigor.

It should also be noted that the findings must be interpreted within the specific organizational and policy framework of the Madrid Regional Health Service (SERMAS). The rapid deployment and sustained integration of teleconsultation at HGUGM were facilitated by pre-existing electronic medical record systems, centralized scheduling infrastructure, and regional mandates promoting non-presential care during the pandemic. These system-level enablers may differ across institutions or countries, potentially limiting the direct generalizability of our results to settings with distinct digital capacities or regulatory structures.

## 7. Conclusions

The shift from 3105 face-to-face visits in 2019 to 2070 teleconsultations in 2020 illustrates an overall 33% reduction but also highlights the rapid adaptation and resilience of the neurosurgical outpatient service at HGUGM. The swift implementation of telemedicine ensured continuity of care despite the near collapse of in-person healthcare services. Although teleconsultation was associated with fewer surgical indications and a modest but statistically significant delay in six-month follow-up, overall adherence exceeded 80%, and non-attendance dropped to zero, demonstrating preserved patient engagement and accessibility.

Five years later, teleconsultation remains integrated into routine neurosurgical workflows, particularly for postoperative reviews and long-term follow-up of stable patients. Its continued implementation within the Madrid Regional Service (SERMAS) and other Spanish tertiary centers reflects the sustained use of non-presential consultation models in the post-pandemic period [41,42].

This sustained adoption confirms that telemedicine is not merely an emergency response but a structural innovation that has redefined the organization of neurosurgical outpatient care.

The HGUGM experience underscores the potential of digital transformation to strengthen healthcare resilience, optimize resource utilization, and extend equitable access to specialized services. The lessons learned from this model—rapid adaptability, hybrid patient management, and long-term continuity—illustrate how telemedicine has become a cornerstone of modern, patient-centered neurosurgical practice.

## Figures and Tables

**Table 1:** Epidemiological data of SARS-CoV-2 infection in Spain during the first epidemic wave. Data from Instituto de Salud Carlos III, 2020 [4].

Region	Cases
Andalusia	15,819
Aragon	6766
Asturias	2690
Madrid	**64,408**
Catalonia	55,196
Castilla-La Mancha	20,477
Castilla y León	23,192
Valencian Community	13,777
Total	**250,273**

Source: Instituto de Salud Carlos III (RENAVE, 2020). Data correspond to confirmed cases from March to May 2020.

**Table 2:** Distribution of patients by year and main consultation characteristics along the two cohorts.

Variable	Total (*n* = 5175)	2019 (*n* = 3105)	2020 (*n* = 2070)	*p*
Age (mean)	59	59	58	*p* > 0.05
Sex (*n*, %): Female Male	2905 (56%) 2270 (44%)	1738 (56%) 1367 (44%)	1167 (56%) 903 (44%)	*p* > 0.05
Month March	2058	1077	981	*p* > 0.05
Month April	1607	996	611	*p* < 0.001
Month May	1510	1032	478	*p* < 0.001
New consultations	795	608	187	*p* < 0.001
Teleconsultations	1203	0	1203	*p* < 0.001

**Table 3:** Consultation types in 2019 vs. 2020 by month.

Type	March 2019	April 2019	May 2019	March 2020	April 2020	May 2020
New	222 (21%)	185 (19%)	201 (20%)	136 (14%)	47 (8%)	4 (1%)
Results	402 (37%)	366 (37%)	384 (37%)	230 (24%)	48 (8%)	42 (9%)
Follow-up	450 (42%)	445 (45%)	447 (43%)	204 (21%)	62 (10%)	65 (14%)
Teleconsultations	0	0	0	382 (40%)	454 (74%)	367 (77%)

**Table 4:** Distribution by pathology and month in 2020.

Pathology	March	April	May	*p*
Location:				
Cranial	58%	31%	11%	*p* < 0.001
Spinal	56%	32%	12%	*p* < 0.001
Other	74%	11%	14%	*p* < 0.001
Type:				
Non-tumoral	46%	30%	25%	*p* < 0.001
Tumoral	60%	28%	11%	*p* < 0.001

**Table 5:** Univariate analysis of variables according to evolution and consultation type.

Variable	By Month *p* and OR (95% CI)		By Consultation Type *p* and OR (95% CI)	
Pathology:				
Tumoral	**<0.001**	0.6 (0.447–0.798)	**0.004**	0.7 (0.508–0.884)
Tumoral benign	**<0.001**	0.5 (0.384–0.760)	**0.014**	0.7 (0.486–0.923)
Tumoral malignant	0.461	0.8 (0.493–1.378)	0.195	0.7 (0.434–1.188)
Vascular	**<0.001**	0.3 (0.203–0.599)	0.314	1.3 (0.791–2.075)
Degenerative	**<0.001**	0.5 (0.437–0.636)	0.810	1.0 (0.811–1.177)
Traumatic	n/a	-	0.398	-
Request for diagnostic tests	**<0.001**	0.5 (0.414–0.681)	0.364	1.0 (0.878–1.426)
Follow-up visits	**<0.001**	0.7 (0.567–0.832)	**0.030**	1.2 (1.021–1.506)
Surgical indications	**<0.001**	0.2 (0.056–0.449)	**<0.001**	0.3 (0.123–0.540)
Delayed 6-month follow-up	0.112	0.8 (0.586–1.390)	**<0.001**	2.0 (1.433–2.741)

Note: Statistically significant values (*p* < 0.05) are in bold. Multivariate model: After adjustment for confounding variables, teleconsultation independently predicted delayed six-month follow-up (aOR = 1.9; 95% CI 1.3–2.8; *p* = 0.002) and a lower likelihood of surgical indication (aOR = 0.4; 95% CI 0.2–0.7; *p* = 0.004). Tumoral pathology remained the strongest independent predictor of surgical decision (aOR = 3.6; 95% CI 2.1–5.9; *p* < 0.001). n/a indicates variables not analyzed due to low frequency (traumatic cases during the study period).

## Data Availability

The datasets analyzed during the current studies are not publicly available due to institutional and GDPR restrictions but are available from the corresponding author on reasonable request.

## Abbreviations

The following abbreviations are used in this manuscript:

aOR	Adjusted odds ratio
CI	Confidence interval
COVID-19	Coronavirus disease 2019
COVIDSurg	COVIDSurg Collaborative
EU	European Union
GDPR	General Data Protection Regulation
HGUGM	Hospital General Universitario Gregorio Marañón
IQR	Interquartile range
N/A	Not Analyzed
OR	Odds ratio
SARS-CoV-2	Severe acute respiratory syndrome coronavirus 2
SD	Standard deviation
SERMAS	Servicio Madrileño de Salud (Madrid Regional Health Service)
WHO	World Health Organization
